# Exploring GPR109A Receptor Interaction with Hippuric Acid Using MD Simulations and CD Spectroscopy

**DOI:** 10.3390/ijms232314778

**Published:** 2022-11-26

**Authors:** Dipendra Bhandari, Sangita Kachhap, Geet Madhukar, Kiran Kumar Adepu, Andriy Anishkin, Jin-Ran Chen, Sree V. Chintapalli

**Affiliations:** 1 Arkansas Children’s Nutrition Center, Little Rock, AR 72202, USA; 2 Jerzy Haber Institute of Catalysis and Surface Chemistry, Polish Academy of Sciences, 30-239 Krakow, Poland; 3 Department of Pediatrics, University of Arkansas for Medical Sciences, Little Rock, AR 72202, USA; 4 Department of Biology, University of Maryland, College Park, MD 20742, USA

**Keywords:** nicotinic acid (niacin), acifran, molecular dynamics simulations, ligand binding, AutoDock, hippuric acid

## Abstract

Previous research has indicated that various metabolites belonging to phenolic acids (PAs), produced by gut microflora through the breakdown of polyphenols, help in promoting bone development and protecting bone from degeneration. Results have also suggested that G-protein-coupled receptor 109A (GPR109A) functions as a receptor for those specific PAs such as hippuric acid (HA) and 3-(3-hydroxyphenyl) propionic acid (3-3-PPA). Indeed, HA has a molecular structural similarity with nicotinic acid (niacin) which has been shown previously to bind to GPR109A receptor and to mediate antilipolytic effects; however, the binding pocket and the structural nature of the interaction remain to be recognized. In the present study, we employed a computational strategy to elucidate the molecular structural determinants of HA binding to GPR109A and GPR109B homology models in understanding the regulation of osteoclastogenesis. Based on the docking and molecular dynamics simulation studies, HA binds to GPR109A similarly to niacin. Specifically, the transmembrane helices 3, 4 and 6 (TMH3, TMH4 and TMH6) and Extracellular loop 1 and 2 (ECL1 and ECL2) residues of GRP109A; R111 (TMH3), K166 (TMH4), ECL2 residues; S178 and S179, and R251 (TMH6), and residues of GPR109B; Y87, Y86, S91 (ECL1) and C177 (ECL2) contribute for HA binding. Simulations and Molecular Mechanics Poisson-Boltzmann solvent accessible area (MM-PBSA) calculations reveal that HA has higher affinity for GPR109A than for GPR109B. Additionally, in silico mutation analysis of key residues have disrupted the binding and HA exited out from the GPR109A protein. Furthermore, measurements of time-resolved circular dichroism spectra revealed that there are no major conformational changes in the protein secondary structure on HA binding. Taken together, our findings suggest a mechanism of interaction of HA with both GPR109A and GPR109B receptors.

## 1. Introduction

G protein-coupled receptors (GPCRs) are one of the largest class of transmembrane proteins used as a therapeutic target. GPCRs are cell surface proteins involved in mediating and regulating wide range of biological processes including immune system, odor, vision, homeostasis, etc. [1,2]. They have also been associated with many disease conditions, such as Alzheimer’s disease, depression, pancreatic cancer, type 2 diabetes mellitus, obesity, cardiovascular diseases, Parkinson’s disease, schizophrenia, and neurological diseases [3,4]. As of 28 October 2022, nearly two-third responses of human hormones and one-third of FDA approved drugs directly involve targeting GPCRs (https://gpcrdb.org), while approximately 500 novel drug candidates targeting GPCRs are in clinical trials [5,6,7]. Human GPCRs can be classified into five different classes—class A (rhodopsin family), class B1 (secretin family), class B2 (adhesion family), class C (glutamate family), class F (frizzled or taste 2) [3]. As of 28 October 2022, out of 826 human GPCRs identified, 165 are validated drug targets and more than 350 have been regarded as druggable (https://gpcrdb.org/structure/statistics) [2]. The function and physiological effect of GPCRs is obtained through their ligand recognition and receptor activation. The activation of GPCRs depends on their endogenous ligands and signals such as amines, peptides, lipids, proteins, small molecules, hormones, neurotransmitters, photons, odors, chemokines, etc. and a variety of intracellular transduction cascades (involving different G-proteins and second messengers) [3,8]. Owing to the pharmacological significance of GPCRs, investigating and exploring the ligands that interact with GPCRs and activates them is of immense importance. 

Interestingly, the G-Protein Coupled Receptor (GPCR)—GPR109A, a class A GPCR, (also known as hydroxycarboxylic acid receptor 2—HCAR2 or HM74A in humans and PUMA g in mice) is expressed in variety of cells and tissue types, more robustly in osteoclastic precursor macrophages [9,10]. GPR109A is now recognized as an important target of niacin (the essential nutrient, vitamin B3 or nicotinic acid) and subsequent interaction of these two molecules led to widespread clinical examinations for the treatment of dyslipidemia and to increase HDL cholesterol [11,12]. Niacin has also been reported to limit lipolysis and hepatic acid accumulation independent of GPR109A without significant metabolic disturbances while fasting [13]. Hippuric acid (HA) which is structurally similar to niacin, and one of the naturally occurring compound belonging to Phenolic Acids (PA) available in blueberry diet has been shown to interact with GPCR—GPR109A inhibiting the process of bone resorption and thereby increasing the bone mass [14]. We therefore discuss the interaction of HA with GPCRs in relation to bone resorption and bone formation. 

Although, importance in bone formation and resorption has mostly been given to micronutrients such as calcium, vitamin D, phosphate and macronutrients comprising fats and proteins [15], recent studies by Chen et al., have shown significantly increased bone formation in rapidly growing male and female rodents when supplemented with a blueberry diet [14]. Specifically, hippuric acid (HA), 3-(3-hydroxyphenyl) propionic acid (3-3-PPA), and PA mixture comprising of all the seven metabolites were found to be potentially bioactive on stimulating osteoblast differentiation and proliferation in cell cultures [14,16]. Gene deletion studies (GPR109A^−^/^−^) in mice, conducted by Chen et al., have revealed significantly higher bone mass and strength in tibia and spine of mice (weaned 4-week-old and 6-month-old) using densitometric, bone histologic, and molecular signaling analytic methods [17]. It was also observed that there is a significant decrease in the several bone resorption markers in serum and bone marrow plasma of GPR109A^−^/^−^ mice. Additionally, in GPR109A^−^/^−^ mice compared with their respective untreated control mice, HA considerably inhibited bone resorption and increased bone mass in wild type mice but had no additional effects on GPR109A^−^/^−^ mice [17]. 

Studies on the structural determinants of GPR109A receptor binding to niacin have been investigated using site-directed mutagenesis by Tunaru et al., revealed putative ligand binding residues in GPR109A receptor, and did not show any interaction of niacin to its close homolog with 95% amino acid sequence identity, GPR109B (also known as hydroxycarboxylic acid receptor 3—HCAR3 and HM74 in humans) [18]. Based on the results of (GPR109A^−^/^−^) knockout studies, site-directed mutagenesis and structural similarity, it can be predicted that the actions of PAs on bone cells are mediated through a mechanism involving specific activation of GPR109A receptor. Other studies involving ligand binding assay (^35^S-GTPγS) using membrane prepared from mouse fetal calvarial cells have shown that HA stimulates ^35^S-GTPγS binding to membranes transfected with GPR109A but not to GPR109B. It has been hypothesized that HA mediates it action on bone cell through binding to GPR109A [19]. Further, results have also confirmed that HA binds to GPR109A similar to niacin but has a weak interaction with GPR109B [19]. 

In the present study, we implemented a computational pipeline involving molecular docking studies, molecular dynamics simulations and Molecular Mechanics Poisson Boltzmann Surface Area (MM/PBSA) calculations [20] combined with in silico mutational analysis and time-resolved circular dichroism spectroscopy to reveal the structural determinants of HA interaction with GPR109A. Taken together, our descriptive and predictive models accord with published findings and support the idea that HA interacts with a higher affinity towards GPR109A than GPR109B, and the residues involved in binding HA to GPR109A are very similar to the ones that mediate binding of niacin molecule. 

## 2. Results and Discussion

**Figure 1:**
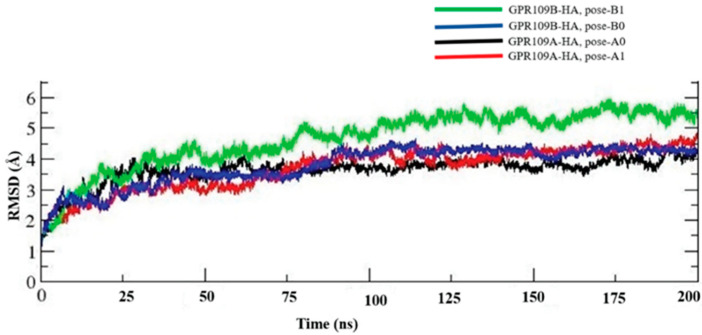
Root Mean Squared Deviations (RMSD) of GPR109A/B-Hippuric Acid (HA) complex.

In the present work, we have performed two separate MD simulation runs on each GPR109A-HA and GPR109B-HA complexes. For both the complexes, the first run (**pose-0**) is the AutoDock predicted top ranked pose with largest population size for both GPR109A and GPR109B structures, whereas the other run (**pose-1**) is the conformation from second largest AutoDock scored cluster. For easy interpretation in the naming pattern, **pose-A0** and **pose-A1** refers to GPR109A-HA complex whereas **pose-B0** and **pose-B1** refers to GPR109B-HA complex. All these complexes were subjected to 200 ns MD simulation followed by trajectory analysis; RMSD based structural clustering of GPR109A/B-HA interaction (Supplementary File S1), and MM/PBSA calculations, which predicts comparative binding energy (not the absolute binding free energy) to check the energetic stability of all the complexes. RMSD plot (Figure 1) of the protein-ligand complex for all four simulations shows the stability of the complex during the simulation.

### 2.1. Interaction of Hippuric Acid with GPR109A

**Figure 2:**
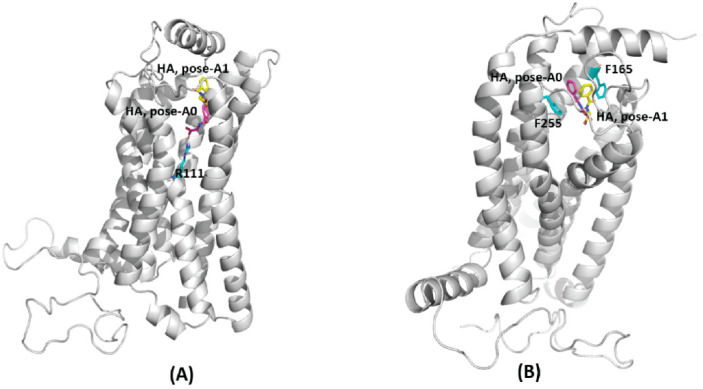
(**A**) Docked structure of GPR109A-HA complex, largest cluster: pose-A0 (purple) and second largest cluster: pose-A1 (yellow). (**B**) Representative structure from the most dominant cluster of GPR109A-HA complex. HA occupies the similar binding site for both the docked poses (pose-A0, purple and pose-A1, yellow) after MD simulation. HA is stacked in between the aromatic rings of F255 and F276.

**Figure 3:**
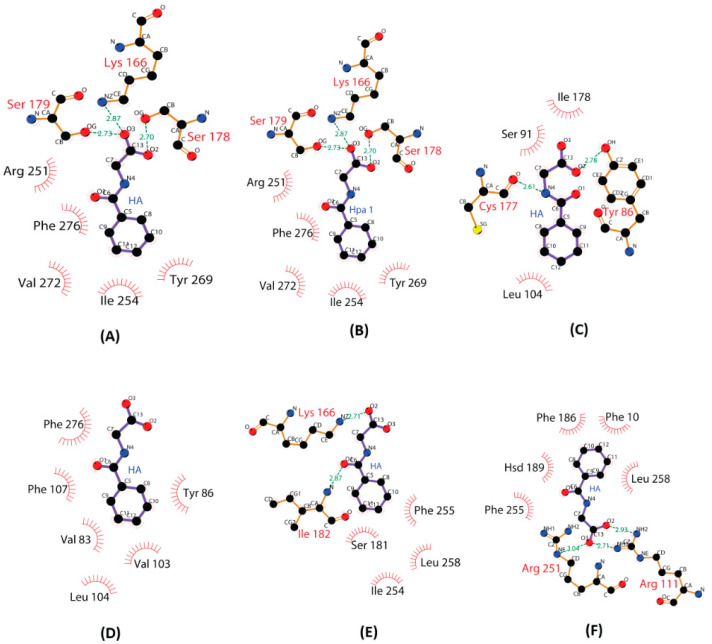
LigPlot representation of GPR109A/B residues interacting with Hippuric Acid (HA) in representative structure from most dominant cluster. All distances are in Å. (**A**) GPR109A-HA, pose-A0 (**B**) GPR109A-HA, pose-A1 (**C**) GPR109B-HA, pose-B0 (**D**) GPR109B-HA, pose-B1 (**E**) Chimera 3A4B-HA (**F**) Chimera 3B4A-HA.

The docked structure of HA with GPR109A in **pose-A0** (Figure 2A) shows HA at a H-bond distance of R111 (a TMH3 residue). During MD simulations, it is observed that HA makes H-bond interaction with TMH4 residue K166, as well as with the ECL2 residues S178 and S179 (Table 1). These interactions of HA with GPR109A residues also can be seen in the LigPlot schematic representation of the representative structure of the most dominant cluster from MD simulation (Figure 3A). Visualization of automated docking through VMD shows that HA aromatic ring is stacked between the aromatic sidechains of F255 (TMH6) and F276 (TMH7) and similar arrangement is also observed in cluster representative structure from MD simulation (Figure 2B). Stacked in between the aromatic rings of F255 (TMH6) and F276 (TMH7), HA makes stable contacts in the binding pocket by forming H-bonds with K166-S178-S179 triad as well as hydrophobic interactions with F255-F276 throughout the MD simulation as detailed in ligplot diagram (Figure 3A).

In **pose-A1**, the conformation from second largest AutoDock scored cluster, HA is docked in the pocket formed by TMH4, TMH5 and TMH6, near to ECL2 without any H-bond interaction with residues in ECL2 region (Figure 2A). During the MD simulations, HA interacts via H-bonds with R111, K166 and R251 (Table 1). However, in the cluster representative structure, HA occupies the similar binding confirmation as **pose-A0** (Figure 2B) making interactions with S178, S179 and K166 (Figure 3B). 

The simulation results have also shown that HA occupies a binding site similar to that of niacin in GPR109A. Comparing HA binding with the results from our earlier studies of niacin [21] and acifran [22] with GPR109A, it is evident that the residues in ECL2 region (S178 and S179) and TMH4 region (K166) along with F255 (TMH6) and F276 (TMH7) are mainly involved in ligand binding. The ligand binding site of GPR109A comprising the residues in ECL2 region (S178, S179) was also experimentally reported in a mutagenesis study by Tunaru et al. [18]. HA being a hydroxyl carboxylic compound and structurally similar to niacin, it is expected to interact in a similar manner and occupy the similar binding site to niacin. 

### 2.2. Interaction of Hippuric Acid with GPR109B 

**Figure 4:**
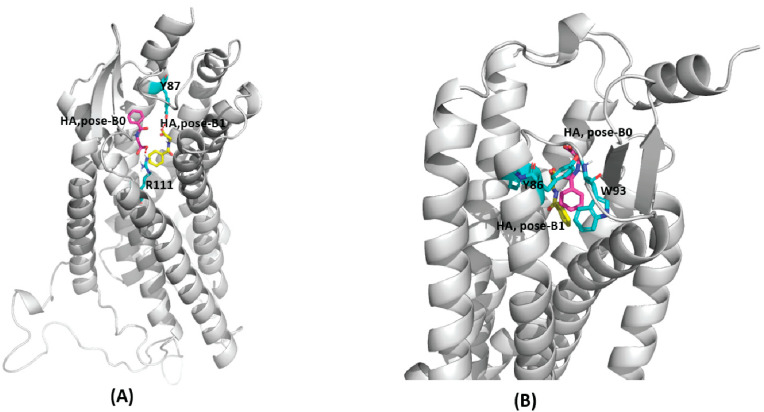
(**A**) Docked structure of GPR109B-HA complex, largest cluster: pose-B0 (purple) and second largest cluster: pose-B1 (yellow). (**B**) Representative structure from the most dominant cluster of GPR109B-HA complex. HA occupies the similar binding site for both the docked poses (pose-B0, purple and pose-B1, yellow) after MD simulation.

In the first complex of GPR109B-HA, i.e., top scored AutoDock predicted model **pose-B0** (Figure 4A), HA is present at the H-bond distance of R111 (TMH3), and the aromatic ring is surrounded by hydrophobic residues of TMH2; V83, Y86, and Y87, ECL1; W93, and TMH3; V103 and L104. However, in the cluster representative structure from MD simulation (Figure 4B), HA makes two H-bonds; one with Y86 (TMH2) through carboxyl group and another with C177 (ECL2) (Figure 3C) and the HA aromatic ring is surrounded by the hydrophobic side chain of residues from TMH2; V83, Y86, ECL1; W93 (ECL1), and TMH3; V103, L104, and F107. H-bond analysis have further shown that, HA makes H-bonds with Y86, Y87 (TMH2), C177 (ECL2) and S91 (ECL1) (Table 2) during MD simulation. 

In **pose-B1**, the conformation from second largest AutoDock scored cluster (Figure 4A), HA carboxyl group makes H-bond with Y87 (TMH2), and the aromatic ring of HA is near to the residues L76 (TMH2) and F107 (TMH3). After MD simulation, in the cluster representative structure from the most dominant cluster, HA occupies similar conformation as that of **pose-B0** (Figure 4B) and forms a similar H-bond profile as that of **pose-B0** (Table 2). Our simulation results have also shown that GPR109B harbors a different binding site for HA than that of GPR109A (Supplementary Files S2 and S3). In GPR109B, HA interacts with the residues from ECL1 (Y86, Y87, S91) and TMH4 (C177) region occupying a binding site in the crevice formed by ECL2, TMH3 and TMH4. 

### 2.3. MM/PBSA Calculation

GPR109A-HA complexes: The calculated binding energies of GPR109A-HA complexes; **pose-A0**, **pose-A1** are −22.77 (±4.67), and −14.96 (±4.87) kcal/mol, respectively (numbers in the parenthesis are standard deviations) (Table 3). The negative energy values suggest that all the complexes are energetically stable. In **pose-A0,** HA is stabilized by forming H-bonds with ECL2 residues K166, S178 and S179, a salt bridge with K166 (TMH4), and hydrophobic interactions with F255 (TMH6) and F276 (TMH7) during MD simulation. However, in **pose-A1**, HA is stabilized by the H-bond formation with R111, R251 and K166. Compared to **pose-A0**, the H-bond occupancy for HA is less in pose-A1 and has weaker binding energy as demonstrated by MM/PBSA energy. 

GPR109B-HA complexes: For GPR109B-HA complexes; **pose-B0** and **pose-B1** the calculated binding energies are −13.52 (±7.07) and −1.48 (±5.78) kcal/mol, respectively (numbers in the parenthesis are standard deviations) (Table 3). In both these complexes, HA is stabilized by the formation of H-bonds with Y87, S91, C177 (ECL2) and Y86 (TMH2). In addition to H-bond, HA is also stabilized by the formation of a salt bridge with R111 (TMH3), and R251 (TMH6). MM/PBSA binding energy values shows that **pose-B1** is energetically less favorable compared to **pose-B0**. 

According to calculated binding energy values, all the complexes of GPR109B-HA are stable. Compared to GPR109A-HA complexes, GPR109B-HA complexes are energetically much less favorable. This is well supported by the finding of the weak binding of HA with GRP109B as observed by Chen et al., [19]. Comparing the MM/PBSA binding energy of GPR109A-HA complex with GPR109A-Niacin (−6.2 ± 5.1 kcal/mol) study from our previous study, GPR109A has higher affinity for HA [21]. 

### 2.4. Chimeric Structure Analysis

The results from H-bond analysis and MM/PBSA calculation have shown that HA has higher binding affinity with GPR109A than that of GPR109B. The major residues that are responsible for binding of HA to GPR109A are present on TMH4 and ECL2—**K166 (TMH4), S178 (ECL2) and S179 (ECL2)** while residues in **TMH2 (Y86, Y87) and ECL1 (S91)** play a major role in binding HA to GPR109B. To understand the importance of the specific residues of GPR109A and GPR109B for binding of HA, we generated two in silico chimeras of GPR109A and GPR109B similar to Tunaru et al., study [18]. Chimera 3A4B consists of first three TM helices including the junction—TMH2/ECL1 from GPR109A and remaining four helices from GPR109B, while in chimera 3B4A, first three TM helices including the junction TMH2/ECL1 are taken from GPR109B and remaining four helices from GPR109A [21]. 

The binding affinity of HA with chimera 3A4B and chimera 3B4A estimated from MM/PBSA calculation is −7.53 kcal/mol (±5.72) and −23.54 kcal/mol (±7.17), respectively (Table 3). MM/PBSA analysis from MD simulations have shown that the affinity of HA decreases for chimera 3A4B than that for GPR109A. This decrease in interaction can be attributed to the loss of one of the major interacting residues S178 to I178 in chimera 3A4B. Additionally, HA has smaller H-bond occupancy with chimera 3A4B as compared to GPR109A (Table 1 and Table 4). The weak interaction is due to the absence of the polar residue S178 and loss of interaction with R251 in chimera 3A4B. In contrast to **pose-A0**, carboxylic group of HA has H-bond interaction with R111 in chimera 3A4B. Absence of residue—S178 in the ECL2 region of chimera 3A4B might have paved a path for HA to move deep inside down the transmembrane helix to interact with R111. The cluster representative structure from MD simulation shows carboxyl group of HA interacting with the side chain of basic residue K166 (Figure 3E). 

The main residues of GPR109B interacting with HA via H-bonds and hydrophobic interaction are in ECL2, THM2 and TMH3 region. For the second chimera 3B4A, which contains first three helices including ECL2 from GPR109B and remaining from GPR109A, MM/PBSA analysis shows that HA has stronger affinity for chimera 3B4A as compared to GPR109B but has comparable binding energy to GPR109A **pose-A0**. Representative structure forms the most dominant cluster for chimera 3B4A shows that HA occupies a binding site surrounded by TMH3 and TMH4 with hydrophobic interaction mainly from F255, H189, F186 and L258 (Figure 3F). Furthermore, HA is stabilized in the binding site of chimera 3B4A by H-bond interaction with basic residues R111, H189 and R251 (Table 5). The presence of 4 helices (TMH4, TMH5, TMH6 and TMH7) along with ECL2 and ECL3 from GPR109A in chimera 3B4A shifted the binding site of HA away from the location in GPR109B. Even in the presence of all the major interacting residues from GPR109B (Y87, C177, Y86, S91, L104) in chimera 3B4A, HA was found to move towards the binding site of GPR109A. This strengthens the finding that HA has stronger binding affinity to GPR109A. The major difference in the amino acid sequence near binding site of GPR109B and chimera 3B4A is the presence of S178 in the later. S178 has been a crucial residue in the binding of HA to GPR109A and similar other small carboxylic acid ligands like niacin and acifran [21,22]. The abrogation of binding for niacin and acifran with GPR109A mutant S178I also explains the importance of S178 in carboxylic acid ligands binding. 

In a mutagenesis study by Tunaru et al., for similar chimeric structures, it was shown that chimera 3A4B was inactive with niacin and had very low binding of EC_50_ (half maximal effective concentration) value greater than 100 µM for acifran [18]. However, chimera 3B4A had stronger interaction with acifran (EC_50_ value ~2 µM) and weak binding with niacin (EC50 value > 100 µM). HA, which is quite larger than niacin in size, but similar to acifran (in terms of size and structure), shows strong interaction with chimera 3B4A similar to that of acifran. This indicates that residues in ECL2 (S178, S179) and TMH3/TMH4/TMH6 (R111, K166, R251) regions are the major contributors in strong binding of HA to GPR109A. 

### 2.5. Free Energy Landscape

**Figure 5:**
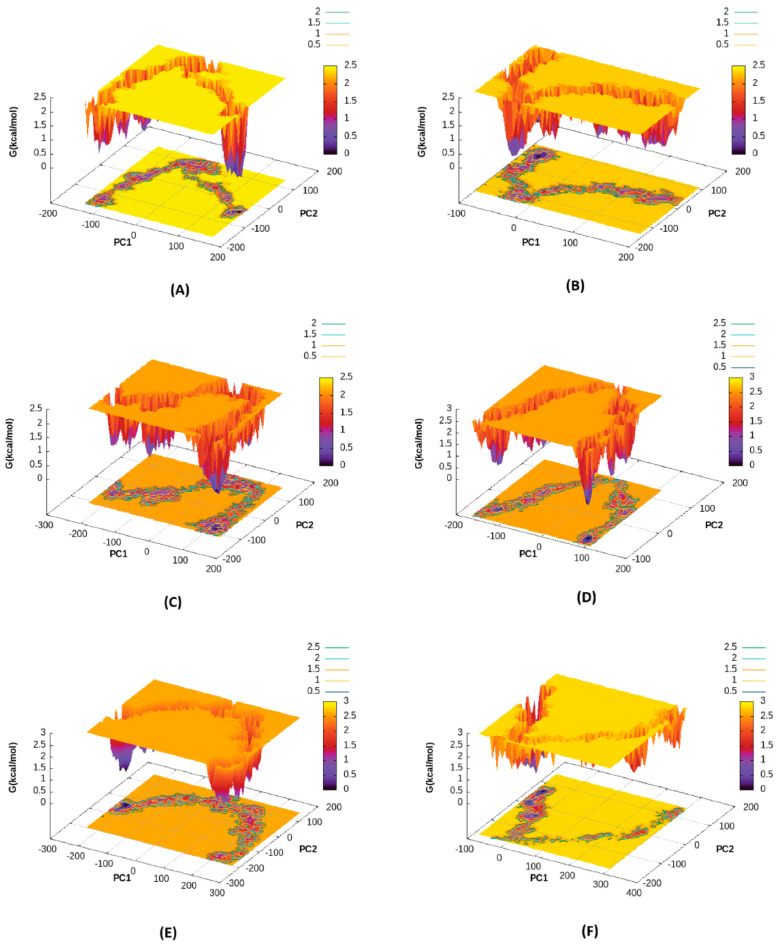
Free Energy Landscape of GPR109A/B—HA Complex, (**A**) GPR109A−HA, pose−A0 (**B**) GPR109A−HA, pose−A1 (**C**) GPR109B−HA, pose−B0 (**D**) GPR109B−HA, pose−B1 (**E**) Chimera 3A4B−HA (**F**) Chimera 3B−HA.

Free energy landscape (FEL) analysis was performed using cpptraj module of AMBER16 [23] to explore the lowest energy conformation obtained during the simulation. A stable protein-ligand system must possess a well-defined energy minimum. In this study, results of FEL for a 200 ns simulation were obtained by mapping Gibbs free energies to the first two principal components—PC1 and PC2 (Figure 5). Principal Component Analysis (PCA) is a method of accessing most significant dynamics by transforming fast local atomic motions from MD trajectories into dominant functional motions. PC1 and PC2 capture the most dominant functional motions from MD trajectories [24]. The energy minimum and energetically favored protein-ligand conformations are represented by dark purple spots in FEL whereas, yellow area represents unfavorable conformations. GPR109A-HA complex, pose-A0 (Figure 5A) shows a distinct energy minimum displaying a stronger binding as compared to all other complexes. Chimeric structures (Figure 5E,F) have a less pronounced energy minima in the FEL representation showing a weak binding of HA.

### 2.6. Circular Dichroism

Time resolved circular dichroism spectra revealed that HA interaction with GPR109A and GPR109B led to small conformational changes in the secondary structure content (α-helix, β-sheet and turns) of the protein (Table 6). A small decrease in the alpha helical content of GPR109A (5.3%) and GPR109B (9.6%) was observed on interaction with HA. Marginal increase in the turns were observed in GPR109A (5.7%) after its interaction with HA, whereas for GPR109B, interaction with HA led to the slight decrease (4.8%) in turns. This slight change in secondary structure by HA binding might be responsible for the activation of GPR109A, as small conformational changes in the secondary structure of GPCRs are associated with its activation [25].

### 2.7. Alanine Mutation Analysis

Since GPR109A-HA complexes are energetically more stable over GPR109B-HA, we considered GPR109A-HA complexes for further in silico mutation analysis to compare the importance of the residues of GPR109A that are interacting with HA for stable binding. Through interaction analysis of GPR109A with HA in MD simulations, we concluded that residues K166 (TMH4), S179 (ECL2), S178 (ECL2) and positively charged residues R111 (TMH3) and R251 (TMH6) are important for HA binding to GPR109A. Additionally, aromatic residues F255 (TMH6) and F276 (TMH7) are also concluded as important residues as they are found to be in the surrounding of HA during MD simulations. Based on these observations, we took the representative structures of the most dominant cluster from MD trajectory of complexes **pose-A0** and **pose-A1** and then subjected the identified critical residues to Alanine mutation. The mutated complex (GPR109A-HA) was energy minimized followed by 50 ns MD simulations for further analysis. 

**K166-S178-S179 Alanine triple mutant:** According to H-bond analysis of **pose-A0** complex, residues; K166 (TMH4), ECL2 residues; S178 and S179 are involved in H-bonding with HA during MD simulation. So based on these results, we generated Ala triple mutants for these residues taking representative structure form the most dominant cluster as the starting structure. Visualization of 50 ns MD trajectory and H-bond analysis both show that due to the Ala mutation HA moved away from TMH4 residue K166A, and ECL2 residues: S178A and S179A during MD simulation. Instead, HA’s carboxyl group made a H-bond with N-terminal residue N17 with occupancy of 20% of the whole MD trajectory. Aromatic ring was surrounded by TMH6 hydrophobic residues; I254, F255, and L258, while in the wild type it is stacked between residues; F255 (TMH6) and F276 (TMH7) (Supplementary File S4). Thus, according to this triple mutant, K166 (TMH4), ECL2 residues S178 and S179 are important for anchoring HA into the binding site similar to our calculations performed on our previous work on niacin binding to GPR109A [21]. 

**R111-R251 Alanine double mutant:** As HA is energetically stable and makes H-bonds with R111 and R251 in **pose-A1,** we generated a double Ala mutant. In the alanine double mutant, HA moved towards ECL2 residues; S178 and S179 during MD simulation and made H-bonds with these residues with an occupancy time of 12% and 19 %, respectively. The HA aromatic ring was surrounded by TMH6 hydrophobic residues: I254, F255, L258, and TMH7 residue F276. Compared to K166A-S178A-S179A triple mutant where HA was unable to reach the arginine binding site, in this specific double mutant, HA was unable to stay near the R111A (TMH3) and R251A (TMH6) due to the absence of any salt bridges. As a result, HA moved towards the ECL2 residues; S178 and S179 (Supplementary File S5). 

**F255-F276 Alanine double mutant:** In complexes **pose-A0** and **pose-A1**, it has been observed that residues F255 (TMH6) and F276 (TMH7) were present near the HA aromatic ring during MD simulations, thus, we considered analyzing the importance of these residues in HA binding to GPR109A. We took the cluster representative structure of two complexes, **pose-A0** and **pose-A1**, to generate the alanine mutant complexes: F255A-F276A-A0 and F255A-F276A-A1 In **pose-A0**, HA makes H-bonds with K166 and ECL2 residues in the wildtype receptor; S178, and S179, and the aromatic ring is stacked between F255 (TMH6) and F276 (TMH7). The H-bond calculation for mutant complex, F255A-F276A-A0, shows that H-bonds of HA with K166, S178 and S179 that were present in wild type complex are also present in the mutant complex with an occupancy time of 17%, 62% and 47%, respectively. Due to alanine mutation of residues F255 (TMH6) and F276 (TMH7), the HA aromatic ring no longer remains in this position during MD simulation and instead shifts to a new site surrounded with ECL2 residues; F186, W188, and H189 (Supplementary File S6). In complex **pose-A1** in WT receptor, the HA carboxyl group interacts with R111 (TMH3), R251 (TMH6), and the aromatic ring is surrounded by hydrophobic residues F255 (TMH6) and F276 (TMH7). In contrast, according to H-bond analysis, HA makes interactions with R111 (TMH3), K166 (TMH4), and R251 (TMH6) with an occupancy time of 57%, 24%, and 52%, respectively, in the F255A-F276A-A1 mutant complex. The HA aromatic ring is surrounded with residues; I254, L258, Y269 throughout the MD simulation. Thus, according to both the mutant complexes (F255-F276-A0 and F255-F276-A1), the hydrophobic residues F255 (TMH6) and F276 (TMH7) in wild type GPR109A are essential to strengthen HA binding (Supplementary File S7). However, as these aromatic side chains are not present in the mutants, HA makes alternate interactions with other residues because of the presence of relatively stable H-bonds and salt bridges despite the absence of hydrophobic interactions with the aromatic residues. 

**K166-S178-S179-R111-R251-F255-F276 Alanine combined mutant:** Finally, we also analyzed the combined alanine mutant of all the above seven residues. To generate the alanine mutant, we considered a frame from MD simulation with **pose-A0** in which the HA carboxyl group made interactions with S178, S179 (ECL2 residue), and the aromatic ring is surrounded by F255 (TMH6) and F276 (TMH7). Visualization of MD trajectory shows that HA moves away from the initially bound position after 4 ns of the MD simulation run, and by the time the simulation reaches the time of 6 ns, HA completely moved away from the initial binding site and exited out from the GRP109A protein (Supplementary File S8). This specific combined mutant confirms the loss of interaction of HA with protein pointing out the importance of these residues (K166-S178-S179-R111-R251-F255-F276) in HA binding. 

## 3. Materials and Methods

### 3.1. Molecular Docking

**Figure 6:**
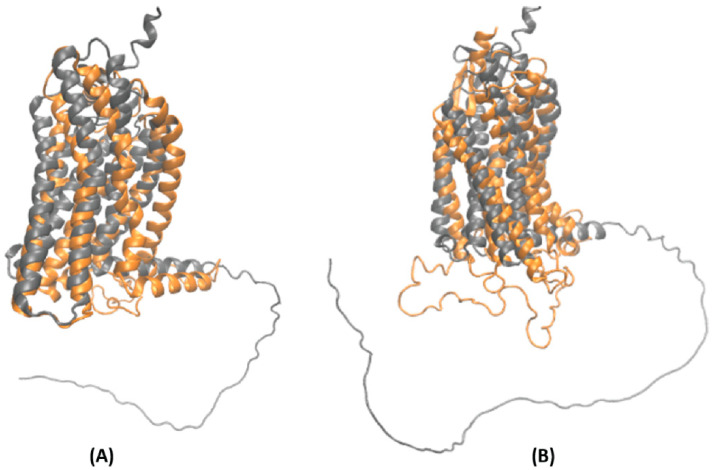
Secondary structure of GPCRs obtained from GPCR-I-TASSER server (orange) and AlphaFold2 database (gray), (**A**) GPR109A/HCAR2 structure (**B**) GPR109B/HCAR3 structure.

**Figure 7:**
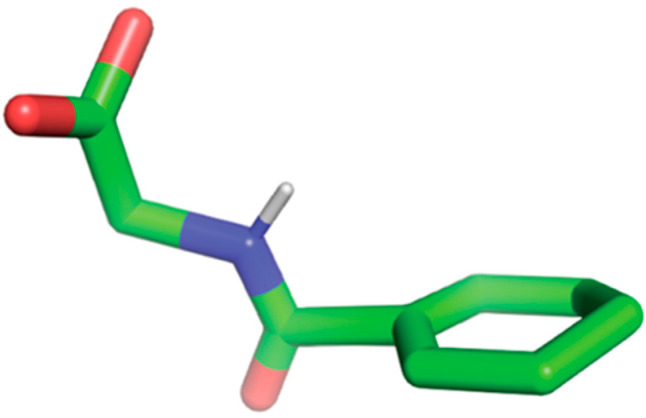
3-Dimensional structure of Hippuric Acid (HA) obtained from PubChem database. Only polar hydrogens are displayed.

The homology model structures for both GPR109A and GPR109B were taken from our previous study of niacin interaction with the GPR109A receptor [21]. Briefly, the primary amino acid sequence of GPR109A/B was submitted to several protein structure prediction servers (GPCR-I-TASSER, Phyre2, SWISS-MODEL and HHpred). The model obtained from GPCR-I-TASSER server had the highest confidence score (C-score) and template modeling score (TM-score); thus, selected for the current study. The comparison of GPCR-I-TASSER modeled structures with GPCRs (hydroxycarboxylic acid receptor 2, HCAR2 and hydroxycarboxylic acid receptor 3, HCAR3) from AlphaFold protein structure database shows a perfect alignment, except for the C-terminus, which is predicted to be highly unstructured. (Figure 6A,B). The initial 3D dimensional structure of HA (Figure 7) for docking studies was retrieved from the PubChem database (https://pubchem.ncbi.nlm.nih.gov/compound/464). AutoDock 4.2 was used to dock HA against GPR109A/B structures and attain initial conformations of the protein-ligand complexes [26]. To explore and facilitate the conformational space of HA in the binding region, the torsional angles for HA and GPR109A/B structures were held flexible during the docking process [26]. Kollman’s united atom partial charges and polar hydrogen atoms were assigned for the proteins using AutoDock tools [26]. As HA closely resembles the niacin structure, we used similar grid box dimensions as mentioned in our previous study with the grid size set to 70 × 70 × 70 points and grid spacing of 0.375 Å [21]. We applied the Lamarckian genetic algorithm (LGA) specified in AutoDock for all the docking calculations. A total 25,000,000 steps of maximum energy evaluation were performed with a population size of 300, while the total number of independent runs was fixed to 150. To group the similar conformations or “clusters” based on their lowest energy conformations and their Root Mean Square Deviations (RMSD) to one another, we used the default clustering algorithm described in AutoDock tools (ADT) [26]. Based on the minimum binding energy, the number of stabilizing interactions such as hydrogen bonds, docked scores, and cluster RMSD values, the final energetically favored docked poses (each separately for GPR109A-HA and GPR109B-HA) were selected as an input for MD simulations. 

### 3.2. Molecular Dynamic Simulations

To get the insights on the interaction and dynamics of GPR109A/B on HA, MD simulations was performed using NAMD starting with the AutoDock predicted protein-ligand conformations [27]. VMD v1.9.3 was used for the assembly of the simulation system, visualization, and analysis of the MD results using custom Tcl scripts [28]. We used pre-equilibrated structure for GPR109A/B embedded into lipid membrane (a mixture of lipids resembling the “generic” plasma membrane of eukaryotes, including 60 cholesterols, 100 DOPC, 20 DPPC, 20 POPA, 60 POPE, and 10 POPS residues) from our earlier studies [21,22]. After insertion of the protein into the lipid bilayer and adding HA, the whole system has been energy-minimized keeping the complex fixed (5000 steps to avoid conflicting contacts, conjugate gradient method) and then simulated with harmonically restrained protein backbone for 10 ns to allow equilibration of the membrane and protein side chains. After that, 200 ns unrestrained MD simulation has followed to explore the dynamics of the ligand. All the MD simulations were performed using NPT ensemble with CHARMM36 force field and TIP3P water model for both GPR109A/B protein and HA molecule [29]. To maintain the electroneutrality of the whole system, a total of 57 K^+^ and 70 Cl^-^ ions were added to GPR109A system and 57 K^+^ and 60 Cl^−^ ions were added to GPR109B system to make up to equivalent of 150 mM salt concentration. During the simulation, Langevin Dynamics was used to maintain constant pressure (1 atm) and temperature (310 K). Periodic boundary settings with a flexible cell were maintained with the cutoff distance of 12 Å for non-bonded interaction and particle mesh Ewald (PME) method was used to treat long-range electrostatic interactions with the switching distance of 10 Å. The coordinates of each system were saved every 1 ps during the entire simulation run. Based on RMSD of GPR109A/B—HA complex throughout the MD simulation, trajectories were extracted and further subjected to cluster analysis (as performed in our earlier study for interaction of niacin with GPR109A) [21]. DBScan algothrim with 25 minpoints (minimum number of points to form a cluster) and a cut-off distance (epsilon) of 0.9 Å in CPPTRAJ module of AMBER16 was used to collect clusters of similar conformations [23]. Representative structure from the largest cluster (Supplementary File S1) was selected for display. To study the binding affinity of HA to GPR109A and GPR109B, we further performed MM/PBSA analysis with the help of café [30], NAMD [27], VMD [28] and APBS [31]. Based on RMSD plot (Figure 3) of the protein-ligand complex and no major conformational changes in the receptors during MD simulation, last 5000 frames with an interval of 10 ps from 200 ns simulation was selected for MM/PBSA analysis.

### 3.3. Construction of Chimeras 

**Figure 8:**
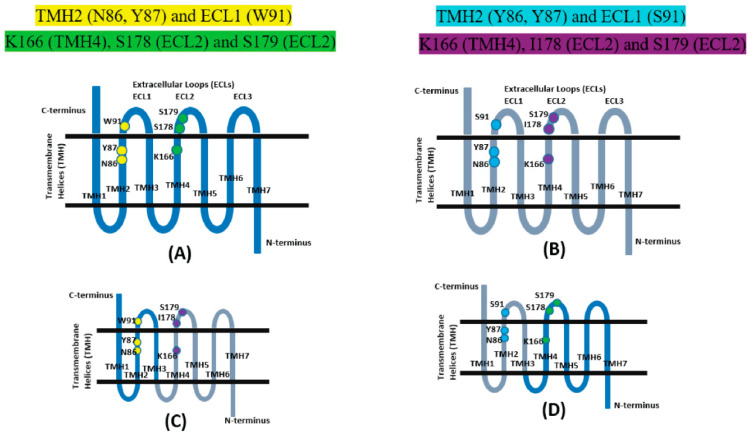
Schematic structure of GPCRs with seven transmembrane helices (TMH) and three extracellular loops (ECLs). Major residues involved in the interaction with HA are highlighted. (**A**) GPR109A, Major residues involved in interaction with HA are highlighted in yellow and green. (**B**) GPR109B, Major residues involved in interaction are highlighted in cyan and purple. (**C**) Chimera 3A4B (first three helices from GPR109A (in blue color) and remaining four from GPR109B (in gray color)). (**D**) Chimera 3B4A (first three helices from GPR109B (in gray color) and remaining four from GPR109A (in blue color)).

Apart from the two AutoDock predicted models, we have generated two in silico chimeras (Figure 8) of GPR109A (Figure 8A) and GPR109B (Figure 8B) from **pose-0** to explore the HA binding site similar to the experimental studies conducted by Tunaru et al. for niacin binding [18]. For the first chimera, three TM helices including the junction—TMH2/ECL1 from GPR109A and remaining four helices from GPR109B are joined to form one chimera, named as 3A4B (Figure 8C). For the other chimera, named as 3B4A (Figure 8D), first three TM helices including the junction TMH2/ECL1 are taken from GPR109B and remaining from four helices from GPR109A. The chimeric constructs were further energy minimized for 10 ns and then subjected to 200 ns MD simulation as explained in methods section.

### 3.4. Circular Dichroism (CD Spectroscopy)

Secondary structural changes in the proteins (GPR109A and GPR109B) after binding to HA was studies by CD spectropolarimeter (J-1500 model, Jasco Instruments, Easton, MD, USA). The recombinant protein (GPR109A and GPR109B) was purchased from MyBioSource (MyBioSource, Inc. San Diego, CA, USA). The lyophilized protein from 20 mM Tris-HCl, 0.15 M NaCl, 0.05% Brij-78 and 6% Treahlose was reconstituted in distilled water to a final concentration of 0.01 µM. Equimolar concentration of HA (dissolved in distilled water) was mixed with the protein. CD spectra were recorded at 37 °C between wavelengths of 190–260 nm in a quartz cuvette of path length 1 mm. Spectra were recorded for protein (only) and for the protein-ligand complex to determine the changes in the secondary structure content. All the analysis were performed using Jasco application software version 1.11.06 [32].

## 4. Conclusions

Structural basis of HA binding to GPR109A based on computational analysis (automated docking, MD simulation, H-bond analysis, and MM/PBSA analysis) clearly revealed that HA has stronger binding with GPR109A, while the binding of HA with GPR109B is comparatively weaker. Our computational results are in agreement with our experimental findings on HA interaction with GPR109A and GPR109B [19]. Furthermore, MD simulations of WT and ALA mutant receptors highlighted important residues of GPR109A for HA binding. Chimeric constructs of GPR109A/GPR109B study strengthens the importance of residues S178, R111 and R251 for HA binding. Drastic reduction in binding energy with chimera 3A4B shows that ECL2 residue S178 is a marker for binding HA. Similar residues have already been found essential for binding of niacin and acifran to GPR109A [18,22]. Additionally, time-resolved circular dichroism spectra showed small conformational changes in the secondary structure content (α-helix, β-sheet and turns) of GPR109A and GPR109B on HA binding. Overall, the results presented here indicate that GPR109A serves as an important potential target for HA binding. Moreover, in the future, experimental techniques such as, bioluminescence resonance energy transfer (BRET) [33], atomic force microscopy based force spectroscopy (AFM-FS) [34], molecular recognition imaging (MRI) [35], isothermal titration calorimetry (ITC) and fluorescence cross-correlation spectroscopy (FCCS) [36], surface plasmon resonance (SPR) spectroscopy [37] can be helpful to study and validate GPR109A/B interactions with HA. 

## Figures and Tables

**Table 1:** Hydrogen bond (H-bond) occupancy of GPR109A–HA complex. Hydrogen bonds are measured with a cut-off distance of 3.5 Å and occupancy times > 5% are displayed in the table.

GPR 109A Residues	H-Bond Occupancy Time (%) in GPR109A-HA Complexes	
	Pose-A0	Pose-A1
**LYS166 (Side Chain)**	73.32	41.98
**SER179 (Side Chain)**	73.06	5.36
**SER179 (Backbone)**	77.88	--
**SER178 (Side Chain)**	41.32	--
**ARG111 (Side Chain)**	--	60.27
**ARG251 (Side Chain)**	--	46.16

**Table 2:** Hydrogen bond (H-bond) occupancy of GPR109B–HA complex. Hydrogen bonds are measured with a cut-off distance of 3.5 Å and occupancy time > 5% are displayed in the table.

GPR 109B Residues	H-Bond Occupancy Time (%) in GPR109B-HA Complexes	
	Pose-B0	Pose-B1
**TYR87 (Side Chain)**	15.79	29.18
**CYS177 (Backbone)**	48.01	15.19
**TYR86 (Side Chain)**	60.12	20.09
**SER91 (Side Chain)**	21.22	13.48

**Table 3:** MM/PBSA calculation for GPR109A/GPR109B-HA complexes.

	* MM/PBSA Binding Energy in kcal/mol (SD)	
	Pose-A0	Pose-A1
**GPR109A-HA**	−22.77 (4.67)	−14.96 (4.87)
**GPR109B-HA**	−13. 52 (7.07)	1.48 (5.78)
**Chimera 3A4B**	−7.53 (5.72)	--
**Chimera 3B4A**	−23.54 (7.17)	--

* MM, Molecular Mechanics; PBSA, Poisson-Boltzmann solvent accessible surface area; SD, Standard Deviation.

**Table 4:** Hydrogen bond (H-bond) occupancy of Chimera 3A4B–HA complex. Hydrogen bonds are measured with a cut-off distance of 3.5 Å and occupancy time > 5% are displayed in the table.

Chimera 3A4B Residues	H-Bond Occupancy Time (%) in Chimera 3A4B-HA Complex
**LYS166 (Side Chain)**	45.34
**ARG111 (Side Chain)**	59.11
**ILE182 (Backbone)**	27.43

**Table 5:** Hydrogen bond (H-bond) occupancy of Chimera 3B4A–HA complex. Hydrogen bonds are measured with a cut-off distance of 3.5 Å and occupancy time > 5% are displayed in the table.

Chimera 3B4A Residues	H-Bond Occupancy Time (%) in Chimera 3B4A-HA Complex
**ARG111 (Side Chain)**	58.25
**ARG251 (Side Chain)**	70.42
**HSD189 (Side Chain)**	38.45

**Table 6:** Secondary structure contents (in %) of GPR109A and GPR109B with and without HA.

	α-Helix	β-Sheet	Turn	Other
**GPR109A**	76.3	0.0	10.2	13.5
**GPR109A-HA**	71.0	0.3	15.9	12.8
**GPR109B**	93.9	0.0	6.1	0.0
**GPR109B-HA**	84.3	0.0	1.3	14.4

## Data Availability

Not applicable.

## Supplementary Materials

The following supporting information can be downloaded at: https://www.mdpi.com/article/10.3390/ijms232314778/s1.

## Abbreviations

G-Protein Coupled Receptor (GPCR), 3-(3-hydroxyphenyl) propionic acid (3-3-PPA), Molecular Dynamics (MD), Extra Cellular Loop 2 (ECL2), Transmembrane Helix 3 (TMH3), Transmembrane Helix 4 (TMH4), Molecular Mechanics Poisson Boltzmann Surface Area (MM/PBSA), Phenolic Acids (PAs), Hippuric Acid (HA), Lamarckian genetic algorithm (LGA) AutoDock tools (ADT), Root Mean Square Deviations (RMSD), Visual Molecular Dynamics (VMD), Circular Dichroism (CD), Free Energy Landscape (FEL), Standard Deviation (SD), Principal Component Analysis (PCA), Circular Dichroism (CD), Molecular Dynamics (MD).
