# Supplementary material for: Exploring GPR109A Receptor Interaction with Hippuric Acid Using MD Simulations and CD Spectroscopy
Source: Int J Mol Sci. 2022 Nov 26;23(23):14778. doi: 10.3390/ijms232314778 (PMC9741133; doi:10.3390/ijms232314778)
Supplement: Supplementary file 1 [file ijms-23-14778-s001.zip › Supplementary File 1.pdf]

Supplementary File 1

Cluster analysis of 200 ns MD trajectory of GPR109A/GPR109B-HA complexes. (A) GPR109A-HA, pose-A0 (B) GPR109A-HA, pose-A1 (C) GPR109B-HA, pose-B0 (D) GPR109B-HA, pose-B1 (E) Chimera_3A4B-HA (F) Chimera_3B4A-HA

(A)

Cluster	Total Frames	Frames Fraction	Average Distance (Å)	Standard Deviation (Å)	Centroid	AvgCDis
0	12739	0.627	5.733	3.103	14103	12.327
1	3769	0.815	6.044	2.537	3015	6.558
2	294	0.014	1.902	0.669	6378	8.824
3	108	0.005	1.936	0.614	6585	6.675
4	85	0.004	2.220	0.700	1313	8.025

(B)

Cluster	Total Frames	Frames Fraction	Average Distance (Å)	Standard Deviation (Å)	Centroid	AvgCDis
0	17722	0.886	6.266	2.742	14981	9.319
1	532	0.027	2.882	1.115	255	6.575
2	176	0.009	2.198	0.646	106	5.489
3	128	0.006	1.760	0.681	856	5.427

(C)

Cluster	Total Frames	Frames Fraction	Average Distance (Å)	Standard Deviation (Å)	Centroid	AvgCDis
0	20094	0.996	8.211	3.872	12847	0.000

(D)

Cluster	Total Frames	Frames Fraction	Average Distance (Å)	Standard Deviation (Å)	Centroid	AvgCDis
0	18650	0.932	6.269	2.669	9322	10.297
1	173	0.009	1.960	0.539	672	10.297

(E)

Cluster	Total Frames	Frames Fraction	Average Distance (Å)	Standard Deviation (Å)	Centroid	AvgCDis
0	8203	0.408	4.269	1.715	16471	10.230
1	7172	0.357	5.055	2.480	995	8.510
2	2357	0.117	4.453	1.718	10080	7.813
3	272	0.014	2.762	1.093	8476	8.334

(F)

Cluster	Total Frames	Frames Fraction	Average Distance (Å)	Standard Deviation (Å)	Centroid	AvgCDis
0	18080	0.904	6.592	2.812	6923	9.584
1	681	0.034	3.189	1.192	916	9.584
