# Supplementary figures and images for: Exploring GPR109A Receptor Interaction with Hippuric Acid Using MD Simulations and CD Spectroscopy
Source: Int J Mol Sci. 2022 Nov 26;23(23):14778. doi: 10.3390/ijms232314778 (PMC9741133; doi:10.3390/ijms232314778)

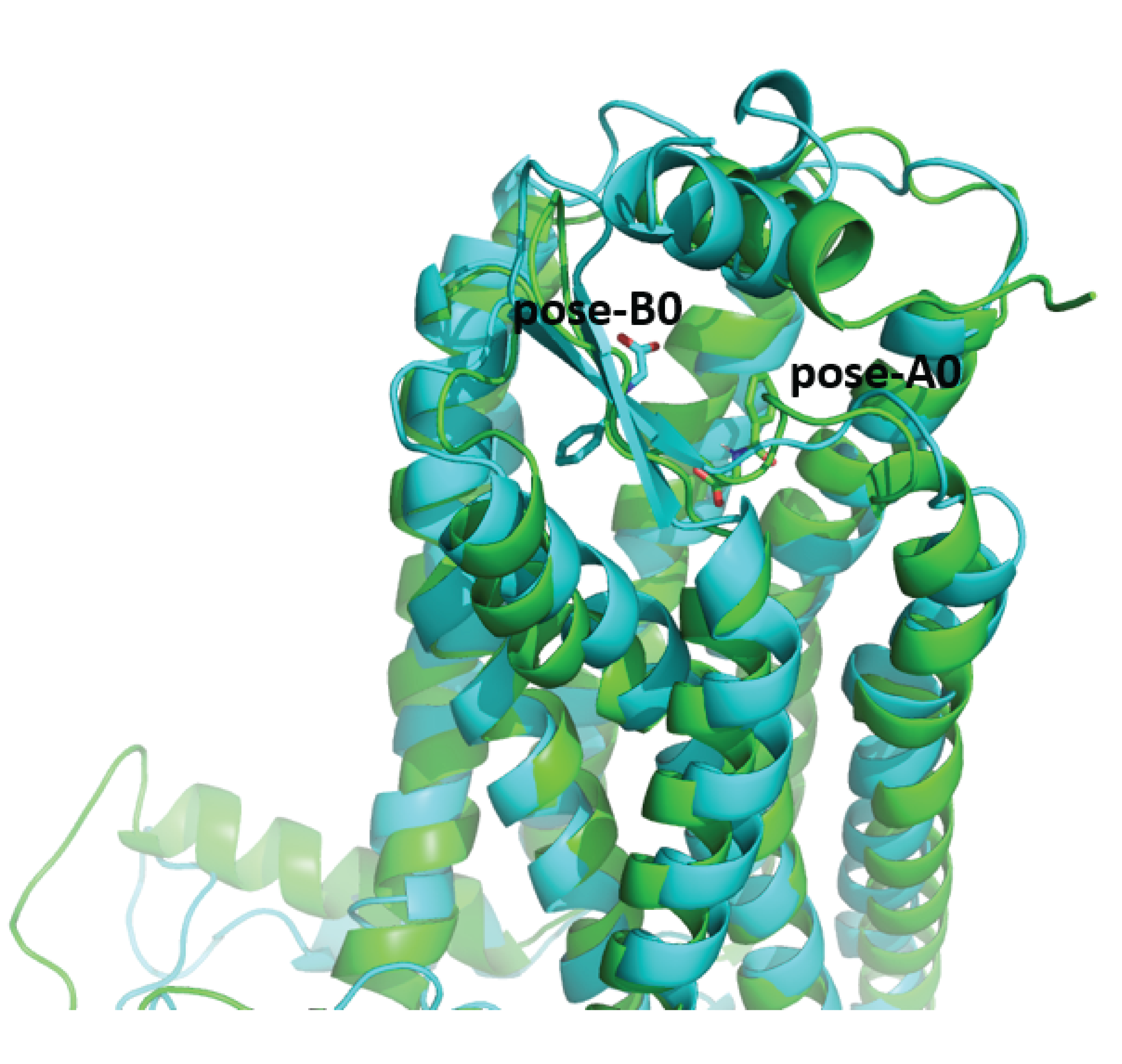

Supplement: Supplementary file 1 [file ijms-23-14778-s001.zip › Supplementary File 2.tif]

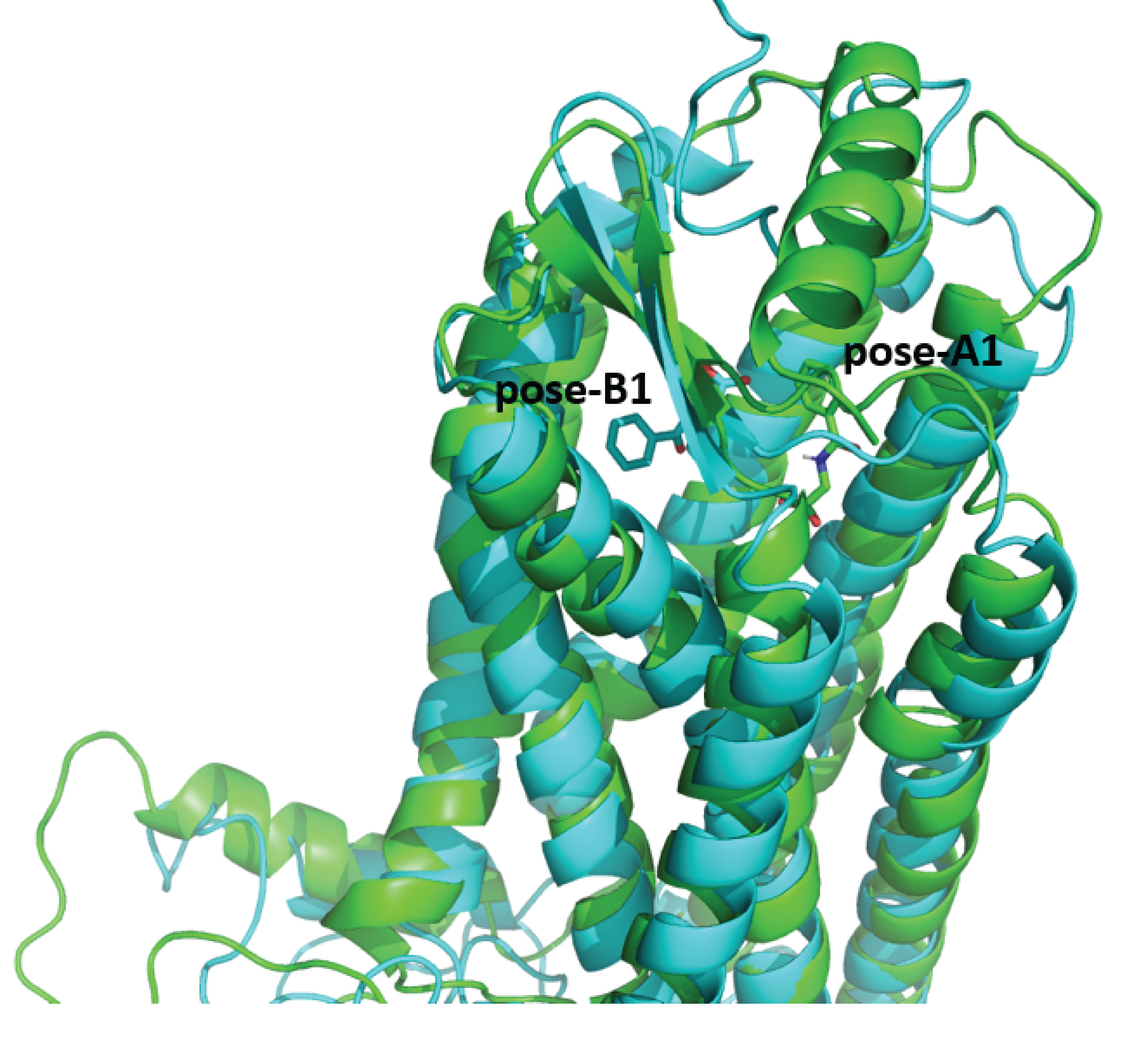

Supplement: Supplementary file 1 [file ijms-23-14778-s001.zip › Supplementary File 3.tif]
